# Design of an Articulated Neck to Assess Impact Head-Neck Injuries

**DOI:** 10.3390/life12020313

**Published:** 2022-02-19

**Authors:** José Luis Rueda-Arreguín, Marco Ceccarelli, Christopher René Torres-SanMiguel

**Affiliations:** 1 Instituto Politécnico Nacional, Escuela Superior de Ingeniería Mecánica y Eléctrica Unidad Zacatenco, Sección de Estudios de Posgrado e Investigación, Mexico City 07738, Mexico; jruedaa@ipn.mx; 2 Laboratory of Robot Mechatronics, Department of Industrial Engineering, University of Rome Tor Vergata, 00133 Rome, Italy; marco.ceccarelli@uniroma2.it

**Keywords:** biomechanics, experimental mechanisms, head-neck impacts, testbed

## Abstract

This paper describes a new solution for an articulated low-cost artificial neck with sensors to assess the effects of head impacts. This prototype is designed as a new solution to evaluate the neck’s response after suffering the head impact. An overview of existing solutions is reported to evaluate the advantages and disadvantages of each one briefly. Problems and requirements for prototype design are outlined to guide to a solution with commercial components. A prototype is developed, and its operating performance is evaluated through a lab test. Several tests are worked out considering the biomechanics involved in the most common accidents of head-neck impacts. Results show a response on the prototype similar to an actual human neck. Future improvements are also outlined for better accurate responses considering the results from the lab test.

## 1. Introduction

Head impacts events are common accidents in daily life activities. These events could lead to mild injuries like traumatism up to severe injuries like different degrees of paralysis or even the death of the person who suffers it [1]. According to the literature, spinal cord injury incidence ranges from 13.1 to 163.4 cases per million persons in developed countries [2,3]. Meanwhile, in non-developed countries, the rates varied from 13.0 to 220.0 cases per million persons [4,5]. The broad ranges might be due to different methods and scopes from the investigation [6]. Several head-neck models can be found in the literature. However, most of these are used in humanoid robots to mimic the basic movements of the head. For example, in Ref. [7], motors and gear belts show four rotational degrees of freedom models. Various head-neck mechanisms were presented in the literature [8]. The first one consists of a wired mechanism, the second one uses a parallel platform with a UPS (Universal-Prismatic-Spherical) configuration, and the last one consists of three degrees of freedom serial manipulator. In most cases where a neck model is developed, the motion is controlled by a user, which means it does not help when analyzing impacts on the head [9].

The most common head-neck model used to evaluate head impacts is the Hybrid III dummy. This dummy consists of a humanoid model with anthropometric dimensions. It is provided with accelerometers and loads cells to obtain data from head impacts in several experimental events [10,11,12]. It is the most Anthropometric Test Device (ATD) used to evaluate vehicle crash events and many other events that could injure a person. Many studies evaluate injuries [13,14,15,16] to mention a few examples: test bench design, which is capable of reproducing impact scenarios by applying a compression force to the thorax produced by the seat belt in a car accident [17], characterization of the impact on the head in terms of acceleration and force, among others [18,19].

Different works like Refs. [20,21,22] show comparisons between Hybrid III and THOR (Test device for Human Occupant Restraint). The mannequins are tested in frontal impacts as reported in Ref. [20] and tension-bending [21]. In Ref. [22], the mannequins are tested under several restraint conditions. THOR mannequin is better in special conditions, such as frontal impacts where airbags do not exist [20]. The THOR mannequin is developed to study specifically impact conditions and has two force cells on the lower and upper neck zone and one accelerometer on the center of the head [23]. In contrast, the Hybrid III mannequin consists of the same cell forces. However, it adds an accelerometer in the neck zone, which is important for acquiring the neck’s acceleration data to evaluate injuries under the HIC (Head Injury Criteria) and NIC (Neck Injury Criteria) criteria. The articulated neck consists of one accelerometer located on the center of the head, one accelerometer on each cervical vertebra model, and cell forces located on each axis of the cervical vertebra model. The force is acting on the neck moment of the impact. The sensors evaluate the injuries on the head and neck through different criteria like HIC and Nij.

In this paper, the design of an articulated neck is presented considering a three cervical vertebra model. The articulated neck is provided with sensors on its vertebras to obtain acceleration and payload data during a head impact event. Performance analysis of the articulated neck model is presented considering three study cases.

## 2. Problems and Requirements

**Figure 1:**
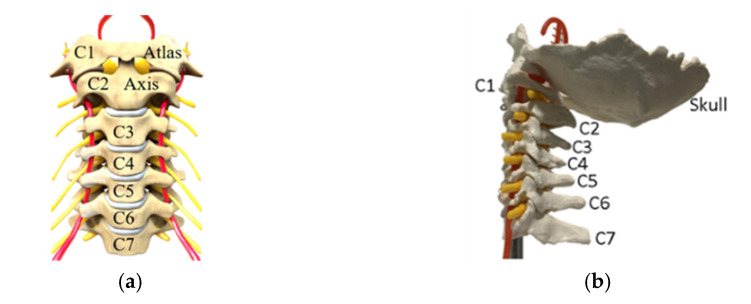
Anatomy of a human neck: (**a**) a scheme; (**b**) market model.

The head and neck are complex biomechanical structures since they are composed of more than twenty muscles and ten bones [24]. The neck consists of the cervical spine, the only part of the vertebral spine involved in the neck motion. It lays between the head and the thoracic vertebrae. It consists of seven vertebras C1–C7, two of which are given unique names, such as C1, known as Atlas, linked between the skull and the C2 in the neck, known as axis, as shown in Figure 1 [25]. Each cervical vertebra has six DOF (degree of freedom). However, many researchers consider the overall neck with three DOF as pitch, roll, and yaw to simplify its motion analysis [26]. Head-neck models like those in Refs. [7,8,9] are used to give the robot’s head movement. However, these models do not acquire any information from the neck.

ATD Hybrid III from Humanetics, Michigan, USA; is the most common dummy used to evaluate human injuries under various circumstances. ATD helps increase the tests’ repeatability and creates a new method to study crash simulations and fatalities. However, ATD is developed based on the human body. In addition, the durability and reliability requirements make an ATD limited in terms of bio fidelity [27]. The neck model in the ATD Hybrid III consists of segmented rubber and aluminum discs with a cable through the center. Additionally, according to the dummy’s specifications, it is found that the instrumentation used for neck evaluations is limited in terms of neck injuries analysis since it consists of only two force cells on the neck located at the upper neck and the lower neck [28].

**Figure 2:**
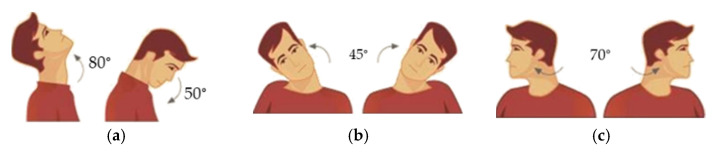
Neck range of movements [12]: (**a**) flexion-extension; (**b**) lateral bending; (**c**) rotation.

Requirements for a suitable articulated neck can be considered with the following aspects:

- An articulated neck structure able to realistically simulate head impact events.
- A model of an articulated neck can consist of at least three cervical vertebrae if the neck motion ranges are fully achieved.
- The neck motions are considered in terms of flexion angles from 50° and extension up to 80°; rotation angles 140° (70° to each side) and lateral bending 45° to each side, as shown in Figure 2 for the complete neck motion range.
- A mechanical design should be defined with low-cost solutions for reasonably easy replacement of broken parts after an impact test, which commonly fails the element tested.

## 3. Modelling and Construction of the Articulated Neck

**Figure 3:**
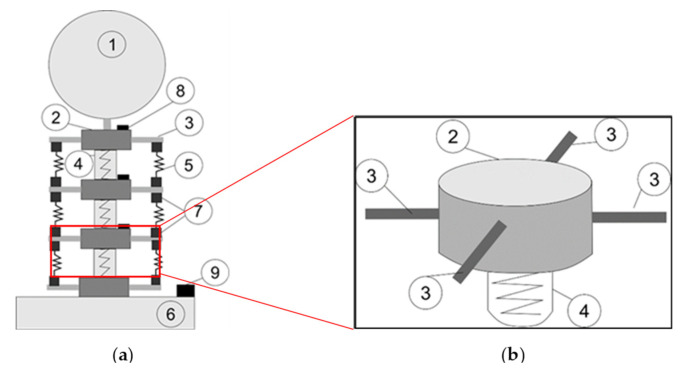
Design of an articulated neck for head impact testing: (**a**) a complete scheme; (**b**) a vertebra segment [29].

A new articulated neck model was designed, taking into consideration a support base (6), three flexible joints (4), three cervical vertebra models (2), eight muscle springs (5), a mannequin head model (1), force sensors (7), and IMU (Inertial Measurement Unit) sensors (8), as shown in Figure 3 [29].

In Figure 3a, the support base (6) is where the articulated neck model is mounted in a prototype solution. It consists of a square shape plastic box of 30 × 30 cm with a height of 7 cm. The base was filled with cement to increase its weight and its stiffness. This element’s main objective is to keep the model in its position and prevent rolling over. The flexible joints (4) are cylindrical aluminum elements. Their sizes are 2.5 cm in height and 1.8 cm in outer diameter. A hole goes through the cylinder to fix the flexible joint to an axis. The flexible joint (4) acts like a metal spring. It can be compressed by 4 mm or extended up to 6 mm. It can also suffer a maximum angular misalignment of five degrees. The motion range from the flexible joint allows to design of an articulated neck in terms of the requirements proposed above. Cervical vertebra models (2) are 3D printed.

**Figure 4:**
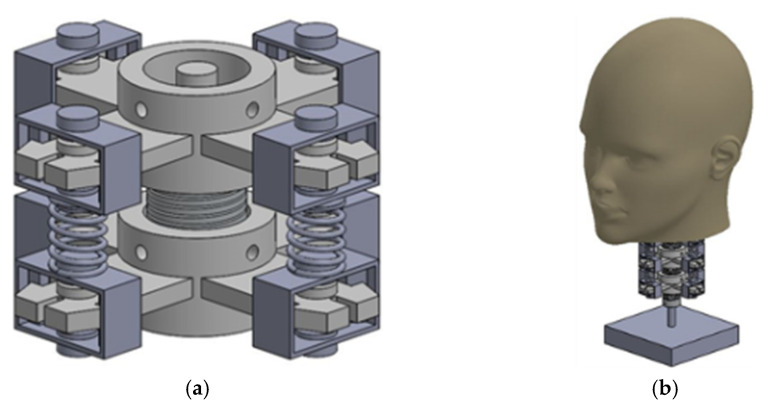
A CAD model of the proposed articulated neck [30]: (**a**) neck model; (**b**) neck with head.

The cervical vertebra (2), shown in Figure 3b, is 3 cm for the outer diameter and 1.9 cm for the cylindrical shape’s inner diameter. Four beams (3) are located on the perimeter of the cervical vertebra, each one is located 90° from the other one. They are defined by rectangular shape dimensions of 4 mm height, 2 cm width, and 4.2 cm longitude. These beams give the neck a larger diameter and allow to place the elements that will simulate the muscles of the neck. Flexible joints (4) are fixed inside the inner diameter of the cylindrical shape of the cervical vertebra model (2). One is placed on each cylinder’s face to create a chain of three cervical vertebrae and three flexible joints, as in the CAD (Computer-Aided Design) design in Figure 4a. Using spring muscles, the beams from one cervical vertebra model (3) are connected to another cervical vertebra model. These spring muscles (5) consist of a metallic spring and a plastic square printed in 3D. The metallic spring sizes are 9.5 mm in outer diameter and 15 mm in height. The square plastic models apply pressure to force cells located on the cervical vertebra beams.

The objective of muscle springs is to simulate the actions of the muscles on the neck. The mannequin head model (1) is fixed to the highest cervical vertebra model. It consists of a polystyrene head model. Weight is added to the head to make it like a real human head. Force sensors (7) are used to measure the force applied by the muscle springs (5). They are placed on the beams of the cervical vertebra model. They are resistive force sensors with a sensor area of 8 mm diameter. Eight force sensors are used to acquire the data from the cervical vertebra’s beams. IMU sensors (8) are used to measure the acceleration of the head impact. Three IMU sensors are used in the prototype. Two IMU sensors are located on the cervical vertebra models, and the last one is located inside the head to measure the acceleration from the center of the head’s mass. Figure 4a shows the assembly of two cervical vertebra models, one flexible joint, and four muscle springs. It is noted that the plastic plates of the muscle springs are attached to the cervical vertebra beam, so they can only move vertically. This is applied to ensure the pressure of the muscle springs against the force sensors. Figure 4b shows a complete prototype design CAD model without sensors [30].

**Figure 5:**
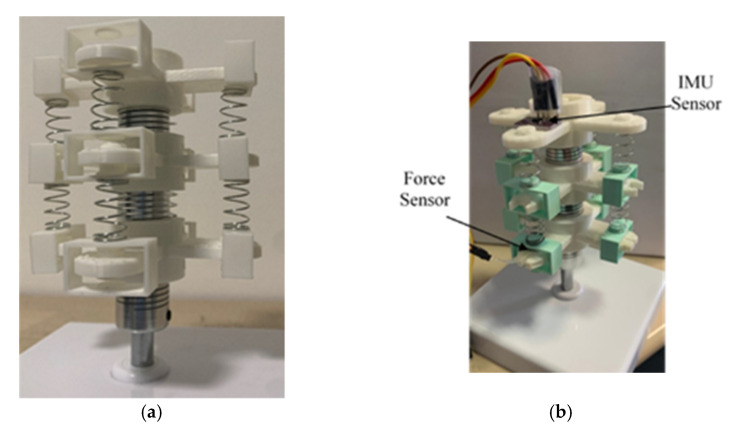
Prototype of an articulated neck: (**a**) main structure; (**b**) articulated neck with sensors.

Figure 5 shows the articulated neck model, whereas Figure 5a shows the prototype assembly of the structural elements of the articulated neck, and Figure 5b shows the location of the sensors on the CAD drawing of the articulated neck. Force and IMU sensors are connected to an Arduino MEGA 2560 to elaborate the sensors’ data acquisition and send them to the user’s computer in force, acceleration, and angular displacement. A low-pass filter was applied during IMUs data acquisition to eliminate noise from the values obtained. This filter also allows to reduce the noise at the moment of sensing the angular displacement.

**Figure 6:**
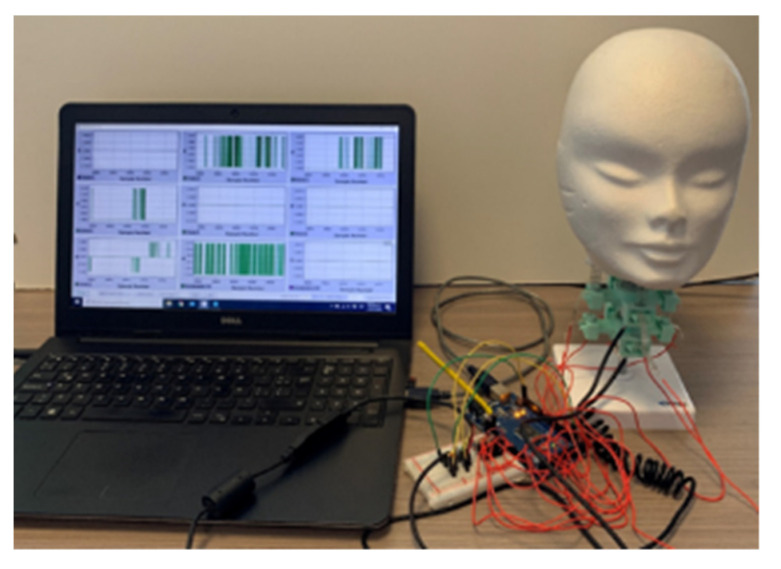
Test layout of a prototype of the articulated neck for analysis performance.

The Java software was used to acquire the data and show the plots in real-time to the user, as shown in Figure 6. A user can save all the information visualized in post-processing and discuss the results.

Figure 6 shows the articulated neck prototype once all the elements are connected in a lab setup. Red wires connect the eight force sensors; USB cables are used on IMU sensors to ensure a good connection.

## 4. Test Modes and Results

The articulated neck is tested considering three different study cases. Case 1 and case 2 consist of a static test. In the first case, the head is bent towards the forward. In the second case, the head is bent towards the right side. Case 3 consists of evaluating the head after a hit on the lateral side of the head. These cases are the most common when a head impact event occurs [18]. The force was applied by using the human force. A user was the one that moved the head on the desired direction.

**Figure 7:**
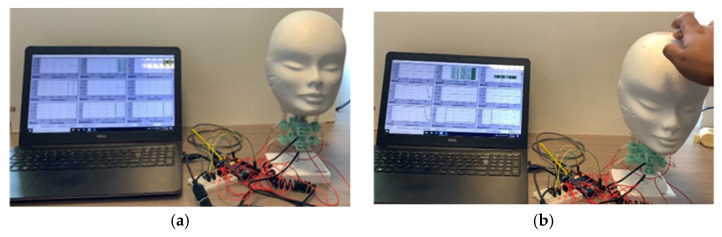
Snapshot of test case 1 for static frontal test: (**a**) at the beginning of the test; (**b**) when applying the force.

The user keeps the head on the desired position for ten seconds and returns it to the initial position for cases 1 and 2. In case 3, a user gives a minor hit on the head model, simulating an impact on the lateral zone. The articulated neck response is evaluated with force applied on the muscle springs, the acceleration and angular displacement on the neck’s cervical vertebras, and the center of the head’s mass. Figure 7 shows the snapshots from case 1. Figure 7a presents the initial position of the head model. Figure 7b shows the head bending towards-forward by force applied by a user.

**Figure 8:**
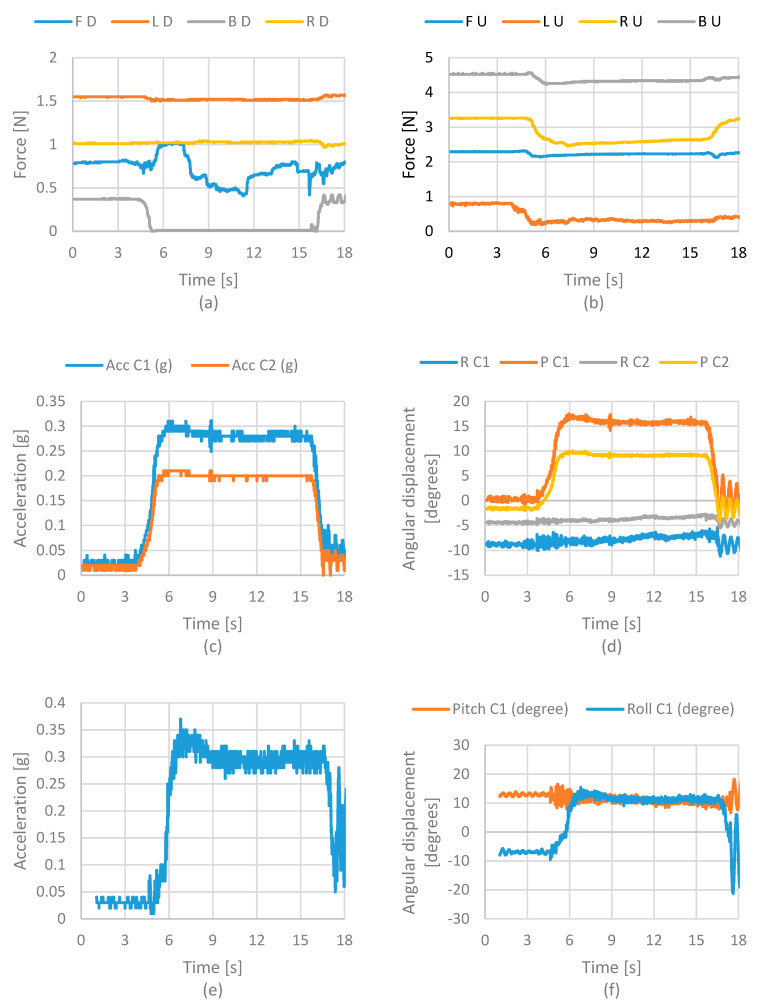
Acquired results from the front static test of case 1 in terms of (**a**) force at lower vertebrae; (**b**) force at upper vertebrae; (**c**) accelerations of the neck; (**d**) angular displacement of the neck; (**e**) acceleration of the head; (**f**) angular displacement of the head. Acronyms: FD—Front Down; LD—Left Down; BD—Back Down; RD—Right Down; FU—Front Up; LU—Left Up; RU—Right Up; BU—Back Up; R—Roll; P—Pitch; C1—Cervical 1; C2—Cervical 2; Acc—Acceleration.

Figure 8 shows the results of force, acceleration, and angular displacements acquired from the sensors. Figure 8a shows the force values from the force sensors on the lower cervical vertebra model. The results show that the sensor’s force on the front suffered an increment on the value while the force sensor on the back reduced its value. These results were as expected. The front muscle spring increases the force to return the head to its original position as the head moves forward. Figure 8b shows the force values from the sensors on the upper cervical vertebra. In this case, also noted is an increment on the front sensor.

The increment is similar to the suffered one on the lower cervical vertebra. This model suffered a significant decrement on the right and left sensor values. This means that muscle springs’ action on the upper cervical vertebra’s sides reduces the pressure when the head bends toward forwards. Figure 8c shows the acceleration values of the cervical vertebras. In this case, the acceleration values represent the movement applied by a user to change the head’s position. The plot shows a small increment on the accelerations on both sensors. Figure 8d shows the angular displacement on the cervical vertebra. These values need to be checked by a user.

**Figure 9:**
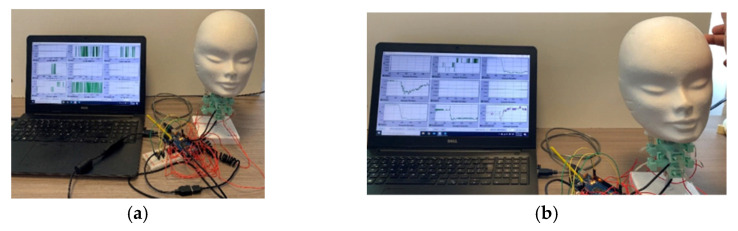
Snapshot of test case 2 for a static lateral test: (**a**) at the beginning of the test; (**b**) when applying the force.

Figure 9 shows the snapshots for case 2. Similarly to Figure 7, Figure 9a shows the initial conditions of the lab test, and Figure 8b shows the snapshot of the test while a user applies the force to bend the head towards its right side.

**Figure 10:**
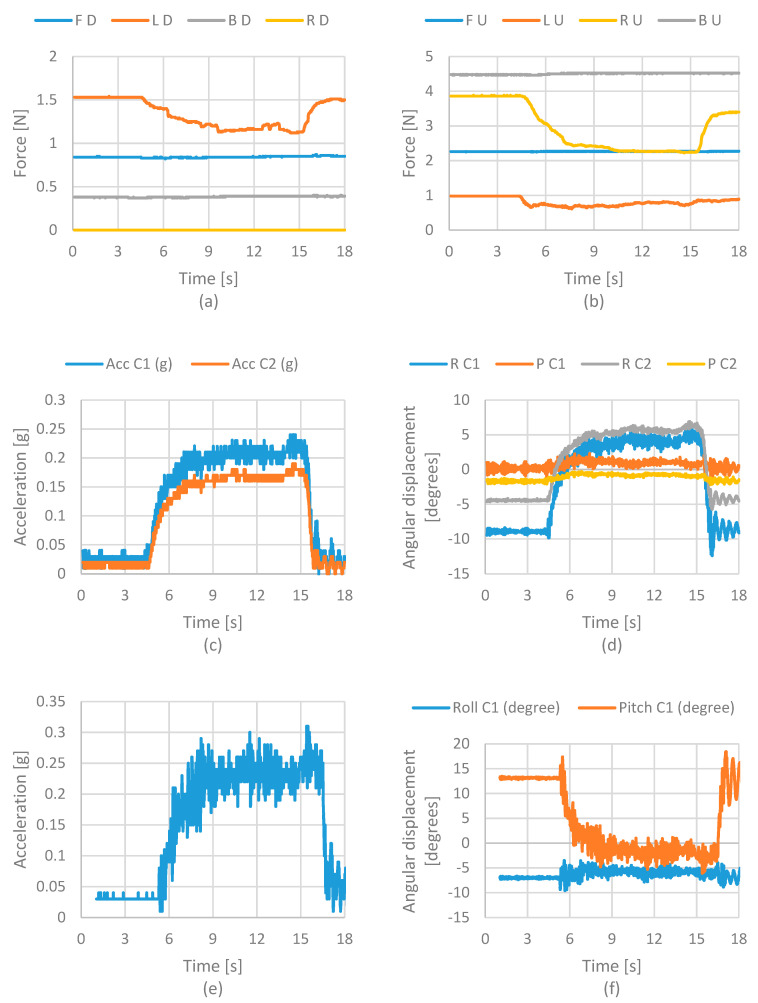
Acquired results from the lateral static test in terms of (**a**) force in the lower vertebra; (**b**) force in an upper vertebra; (**c**) accelerations on the neck; (**d**) angular displacement on the neck; (**e**) acceleration on the head; (**f**) angular displacement on the head. Acronyms: FD—Front Down; LD—Left Down; BD—Back Down; RD—Right Down; FU—Front Up; LU—Left Up; RU—Right Up; BU—Back Up; R—Roll; P—Pitch; C1—Cervical 1; C2—Cervical 2; Acc—Acceleration.

Figure 10a shows force at the lower cervical vertebrae when the head bends toward its right side. It is noted that the front and rear sensors sense the same force value in this case. A sensor shows a decrement of the value, but then it is increased as expected. Figure 10b shows the results from the upper cervical model. In this case, the results are similar to the lower cervical model. The front sensor does not change its values, and the left and right sensors show a decrement and an increment, respectively. The acceleration of the cervical models is shown in Figure 10c. In this case, the result is similar to case 1. The sensor does not show a considerable variation because the user is the one that moves the head. In case 3, a lateral head impact is evaluated.

**Figure 11:**
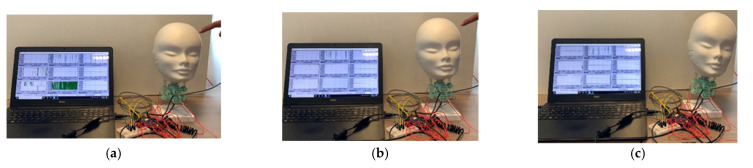
Snapshot of test case 3 for lateral impact test: (**a**) at the beginning of the test; (**b**) when applying the force; (**c**) end of the test.

Figure 11 shows the snapshots of the performed test. Figure 11a shows the test’s initial conditions, Figure 11b shows the impact of the user’s force, and Figure 11c shows the head model after the impact.

**Figure 12:**
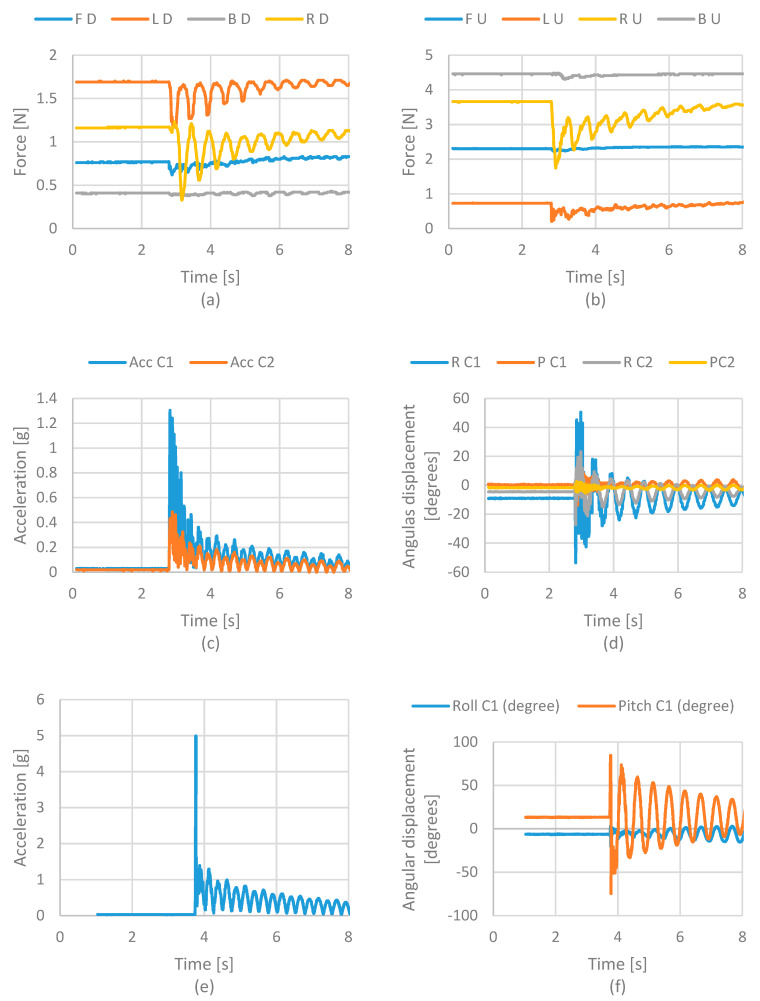
Acquired results from lateral impact test in terms of (**a**) force in the lower vertebra; (**b**) force in an upper vertebra (**c**) acceleration on the neck; (**d**) angular displacement on the neck; (**e**) acceleration on the head; (**f**) angular displacement on the head. Acronyms: FD—Front Down; LD—Left Down; BD—Back Down; RD—Right Down; FU—Front Up; LU—Left Up; RU—Right Up; BU—Back Up; R—Roll; P—Pitch; C1—Cervical 1; C2—Cervical 2; Acc—Acceleration.

The results show oscillation due to deformation of the springs and flexible joints when the head’s movement is experienced after impact. Figure 12a shows the results regarding the force of the lower cervical vertebra model. It can be noted that the force cell located on the left side is the one that has a more significant variation with values from 0 to 0.8 N. The force cell located on the right side also has slight variation, but in a range that goes from 0.8 to 1 N. Front and rear force cells present a variation from 0 to 0.1 N. Figure 12b show more significant force acting on the right and left sensors, while the front sensor does not change its values. Figure 12c displays the acceleration of cervical models. The plot shows that the upper cervical model has a more significant acceleration. The lower cervical vertebra model is closer to the fixed point.

Considering the results from the impact test in Figure 12, it is possible to estimate the HIC (Head Injury Criteria) and the Nij (Neck Injury Criteria). HIC consists of evaluating the acceleration on the head at the moment of the impact. For this, the acceleration of the head model is used. This value is obtained from the IMU sensor located on the center of mass of the head model.

The HIC evaluates the possible injury on the head considering the acceleration suffered at the moment of the impact. The formula to calculate the HIC is the following one:

(1) HIC=max{1(t2−t1)32[∫t1t2a(t)dt]52}

**Figure 13:**
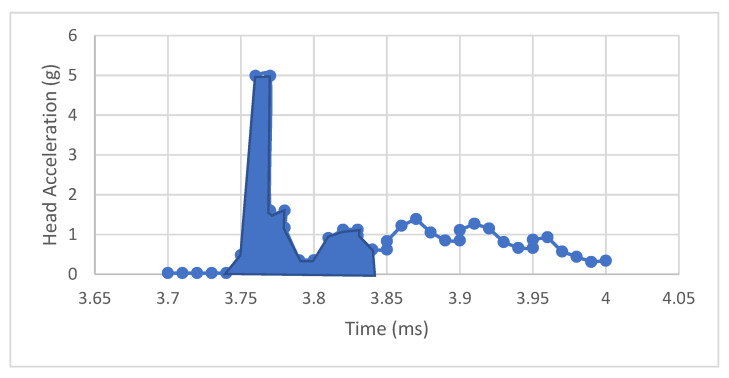
The HIC Test.

HIC evaluates the acceleration of the head in a 0.36 s. Figure 13 shows the interval where the HIC is evaluated for this case. In Table 1, HIC values from tests performed are presented.

Note that the HIC index value is small due to the force used to generate the impact. Also, it is essential to note that the IMU sensors acquired information on the period required to evaluate the HIC, which was 0.36 s.

Additionally, it is possible to evaluate the Nij. This criterion evaluates the force and momentum on the neck at the impact. This information is obtained from force sensors on each cervical vertebra of the designed articulated neck.

(2) $$\mathrm{Nij}=\frac{F_{z}}{F_{\mathrm{int}}}+\frac{M_{y}}{M_{\mathrm{int}}}$$

**Figure 14:**
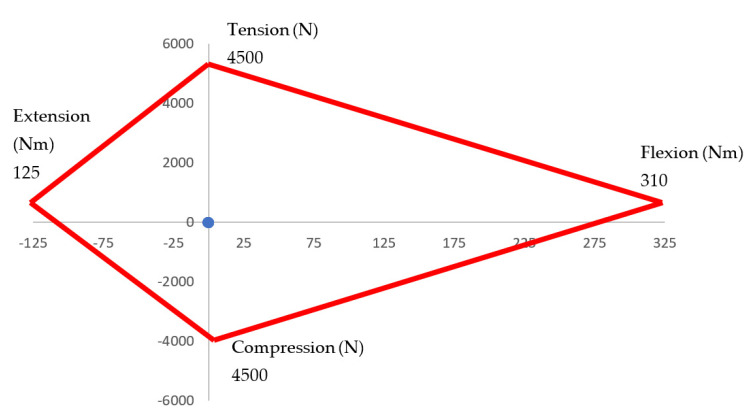
Nij from Impact test.

The values F_int_ and M_int_ are critical load values established by the NHTSA (National Highway Transportation Safety Administration) for the maximum axial load in tension or compression and the measured flexion or extension bending moment established by NHTSA [31,32]. The values F_z_ and M_y_ are calculated from the results from the experimental tests. F_z_ is obtained by multiplying the mass of the head by the acceleration obtained from the experimental tests. M_y_ is a function depending on Fz multiplied by the angular distance the head moves measured by the accelerometers during the tests. Figure 14 shows the results from the impact tests for the Nij Index. Also, it is possible to observe the limits of the index criteria. These limits indicate the maximum momentum (flexion and extension) and force (tension and compression) a neck resists without injury. It is noted that the results of the tests are small and that they are only visible as a dot on the graphics. These results were expected due to the type of force applied to generate the impact.

Finally, the motion response for the articulated neck was previously evaluated in Ref. [30], where the authors present a simulation of the articulated neck. The motion results are compared to confirm the motion range between the prototype proposed and an actual human neck. Force sensors located on each cervical model are constantly under pressure. The force is measured considering the force applied when the cervical get closer on each road, and the sensors measure the increment of the force.

## 5. Discussion

The articulated neck presented shows a prototype implemented for lab test experiments to evaluate head impacts. The performance and motion response to the force applied to mimic the response of a human neck. The articulated neck results are evaluated on head impact events similar to those obtained by the authors [30]. The force’s application needs to be controlled and to have the same magnitude in all the experiments performed. Using an operator’s hand to perform the impact does not assure the experiment’s repeatability. It is important to apply the force or impact by using a mechanism or a testbed that can measure the magnitude of the force and give the same magnitude in all the tests performed. IMU sensors are located on each cervical vertebra to sense the acceleration and angular displacement that results in the articulated neck’s biomechanical response.

The location of the IMU tries to be the closest to the center of mass of each vertebra. The work shows that the force sensors on each cervical vertebra measure the force applied at the moment of the impact. The prototype can simulate low-speed head impact, as shown in the results. Following the development of the prototype, it is important to evaluate the articulated neck on the head impact testbed like the one presented in Refs. [33,34] by the authors.

## 6. Conclusions

The reported lab experiences successfully characterize the proposed articulated neck’s response regarding the forces suffered on each cervical vertebra model. During tests for cases 1 and 2, the muscle springs show head movement as a natural human activity, while case 3 shows the neck muscles’ response with significant values after receiving an impact. The data acquisition in terms of force from each cervical will help understand the cervical force and moments that can lead to severe injuries from a better specific point of view. The articulated neck increased the number of sensors in the neck zone. This helps to evaluate better the forces applied on that zone at the moment of suffering an impact. As shown in cases 1, 2, and 3, the neck can evaluate the neck’s forces when applied to the head. Flexible joints and springs help to simulate the biomechanical response of the neck. The mechanism mimics the neck’s motion ranges, considering the six DOF in each cervical vertebra. Springs are also the elements that simulate the muscle response to return the neck to its initial position. The articulated neck evaluates the injuries using the Nij; this criterion is based on the impact between the moments and the force acting on the neck. The paper shows that the articulated neck prototype permits measuring the force magnitude on each cervical vertebra.

Additionally, the moments can be calculated easily, considering the geometry of the cervical model. The authors considered improving spring elements to increase the articulated neck’s stiffness for future works. Authors will also combine the research of this work with the application of a testbed to evaluate head impacts as reported in Ref. [34] to increase the biofidelity of the head impacts analysis. Finally, this research motivates future efforts to design equipment to prevent head-neck injury [35].

The analysis of the head-neck model by different injury criteria allows to observe an advantage within the designed model, which allows the analysis of injuries by different criteria by acquiring the necessary information for the evaluation of the different head and neck injury criteria.

## 7. Patents

Collo artificiale articolato per testa di manichino “Artificial Articulated Neck for Mannequin tests”, number 102020000005596, submitted 16 March 2020.

## Figures and Tables

**Table 1:** Shows the HIC injury criteria from 5 impact tests performed.

Impact Test	HIC Value
1	0.298
2	0.311
3	0.294
4	0.305
5	0.302

## Data Availability

Not Applicable.
